# Ankaferd Influences mRNA Expression of Iron-Regulated Genes During Iron-Deficiency Anemia

**DOI:** 10.1177/1076029617737838

**Published:** 2017-11-07

**Authors:** Afife Gulec, Sukru Gulec

**Affiliations:** 1 Molecular Nutrition and Human Physiology Laboratory, Food Engineering Department, İzmir Institute of Technology, Urla, İzmir, Turkey

**Keywords:** Ankaferd Blood Stopper, iron, iron-deficiency anemia, Caco-2, HepG2, gene regulation

## Abstract

Ankaferd Blood Stopper (ABS) comprises a mixture of plants and stops bleeding via forming a protein network by erythroid aggregation. Bleeding causes reduction of iron levels in body. It has been indicated that ABS contains significant amount of iron. Thus, we investigated the biological activity of ABS-derived iron on iron-regulated genes during iron-deficiency anemia (IDA). IDA We selected Caco-2 and HepG2 cell lines as in vitro models of human intestine and liver, respectively. Iron deficiency anemia was induced by deferoxamine. The cells were treated with ferric ammonium citrate (FAC) and ABS. Messenger RNA levels of iron-regulated genes were analyzed by quantitative reverse transcription polymerase chain reaction to elucidate whether iron in ABS behaved similar to inorganic iron (FAC) during IDA. The results showed that ABS-derived iron influenced transcriptions of iron-regulated marker genes, including divalent metal transporter (*Dmt1*), transferrin receptor (*TfR*), ankyrin repeat domain 37 (*Ankrd37*), and hepcidin (*Hamp*) in IDA-induced Caco-2 and HepG2 cells. Our results suggest that when ABS is used to stop tissue bleeding, it might have an ability to reduce levels of IDA.

## Introduction

Ankaferd Blood Stopper (ABS) is commonly used to stop bleeding of tissues.^1,2^ It has been shown that ABS reduces the growth rate of pathogens indicating its protective effect against microorganisms.^3^ Furthermore, ABS enhances bone cell growth and regeneration in vivo.^4,5^ It also affects the levels of transcription factors in human vessel endothelial cell model.^6^ These studies indicate that ABS has effect on the physiology of cells by influencing molecular and genetic mechanisms. It has been recently reported that ABS contains iron.^7^ Iron is one of the important transition metals in human and involves in variety of physiological functions including oxygen carrying, cell division, inflammation, and energy production.^8^ Consuming low levels of dietary iron or bleeding lead to iron-deficiency anemia (IDA) in human, whereas high iron in human body causes iron toxicity in tissues (hemochromatosis).^8,9^ Dietary iron absorption mainly occurs via enterocyte cells on duodenum and upper jejunum of small intestine. Intestinal iron absorption is essential to maintain iron balance in human body because human does not have active excretion mechanism for iron.^10^ Iron can be stored in liver or used by bone marrow to make more red blood cells. Mature red blood cells can be broken down by macrophages of spleen and iron is released into blood circulation.^8^ Iron metabolism is tightly controlled by the molecular and genetic mechanisms. Low iron (iron deficiency) and hypoxia are 2 main physiological signals for intestinal iron absorption and iron metabolism.^11^ Iron deficiency leads to induction of iron transporter genes and this is an important adaptation of cell to absorb more dietary iron into the body.^12,13^ When iron demand is low, induction of iron-regulated genes can reach normal physiological levels. Systemic iron level is controlled by hepcidin protein (HAMP) that is secreted from the liver. The HAMP leads to ferroportin 1 protein degradation during elevated iron level in human body.^14^

The recent investigation has shown that ABS has significant amount of iron.^7^ This observation let us hypothesize that iron in ABS solution might have ability to reduce IDA. We tested this idea in iron-deficiency-induced Caco-2 and HepG2 cell lines by analyzing expression of iron-regulating genes. We showed that iron in ABS solution was able to reduce IDA in vitro.

## Material and Methods

### Cell Culture

Caco-2 cells were maintained in Eagle’s minimum essential medium supplemented with 20% fetal bovine serum (FBS), 1% nonessential amino acids, 1% sodium pyruvate, and 1% penicillin and streptomycin. Same medium mix was used for HepG2 cells with 10% FBS. Cells were maintained and grown in 5% CO_2_ incubator at 37°C. Passage numbers of the cells were kept between 20 and 35. These cells have been commonly used as an in vitro model in human intestine and liver iron physiology.^15,16^ Experimental Caco-2 and HepG2 cells were plated in 12 wells and grown for 21 and 5 days postconfluent, respectively. Cell culture medium was changed every 2 days until deferoxamine mesylate (DFO), ABS, and ferric ammonium citrate (FAC) treatments were performed.

### Iron Measurement From ABS

The ABS was provided by Dr Haznedaroglu (Department of Hematology, Faculty of Medicine, Hacettepe University, Ankara, Turkey). About 10 mL ABS was centrifuged at 100*g* for 5 minutes. The ABS samples were utilized for acid digestion, and atomic absorption spectroscopy was used to measure iron levels in ABS solution.^17^

### Induction of Iron Deficiency and Treatments of FAC and ABS

Deferoxamine mesylate 100 µM was used for 24 hours to induce IDA in both cell types. Cells were treated by 100 µg/mL FAC, and FAC was used as control group for experimental conditions to compare ABS-derived iron on genetic regulation of cells. Both cell types were exposed to same amount of iron-containing FAC and ABS solutions with same volume.

### RNA Isolation and Quantitative Reverse Transcription Polymerase Chain Reaction

RNA isolation was performed by RNAzol reagent (MRC Inc., OH, USA) according to the company’s instruction. Total RNA sample 1 µg was used to make complementary DNA synthesis (Life Technologies, California), and quantitative reverse transcription polymerase chain reaction (RT-qPCR; Life Technologies) was performed by following company protocols. Gene-specific primers were used for iron- and hypoxia-regulated genes (Table 1). Cyclophilin A1 (*CypA1*) was chosen as a housekeeping gene to normalize the data. The RT-qPCR data were converted to gene expression levels via 2^ΔCt^ calculation methods.^16^

**Table 1.** Primer List

Gene	Forward (5′ to 3′)	Reverse (5′ to 3′)
*CypA1*	TACGGGTCCTGGC ATCTTG	CGAGTTGTCCACA GTCAGCA
*Dmt1*	TGCATCTTGCTGA AGTATGTCACC	CTCCACCATCAGC CACAGGAT
*TfR*	TCAGAGCGTCGG GATGATATCGG	CTTGATCCATCATC ATTCTGAACTGCC
*Ankrd37*	AGCAGTCGCCTGT CCACTTAGC	AGCAGGCTTAGGC ACTCCAGG
*Hamp*	AGTGGGACAGCCA GACAGACG	CAGCTCTGCAAGTT GTCCCGT

### Statistical Analyses

All results were expressed as mean (standard deviation). One-way analysis of variance with post hoc Tukey test was performed for relative gene expression to compare experimental groups. GraphPad Prism (version 6.0 for Windows; GraphPad. CA,USA) was used to create all the figures and for statistical analysis.

## Results

**Figure 1.**
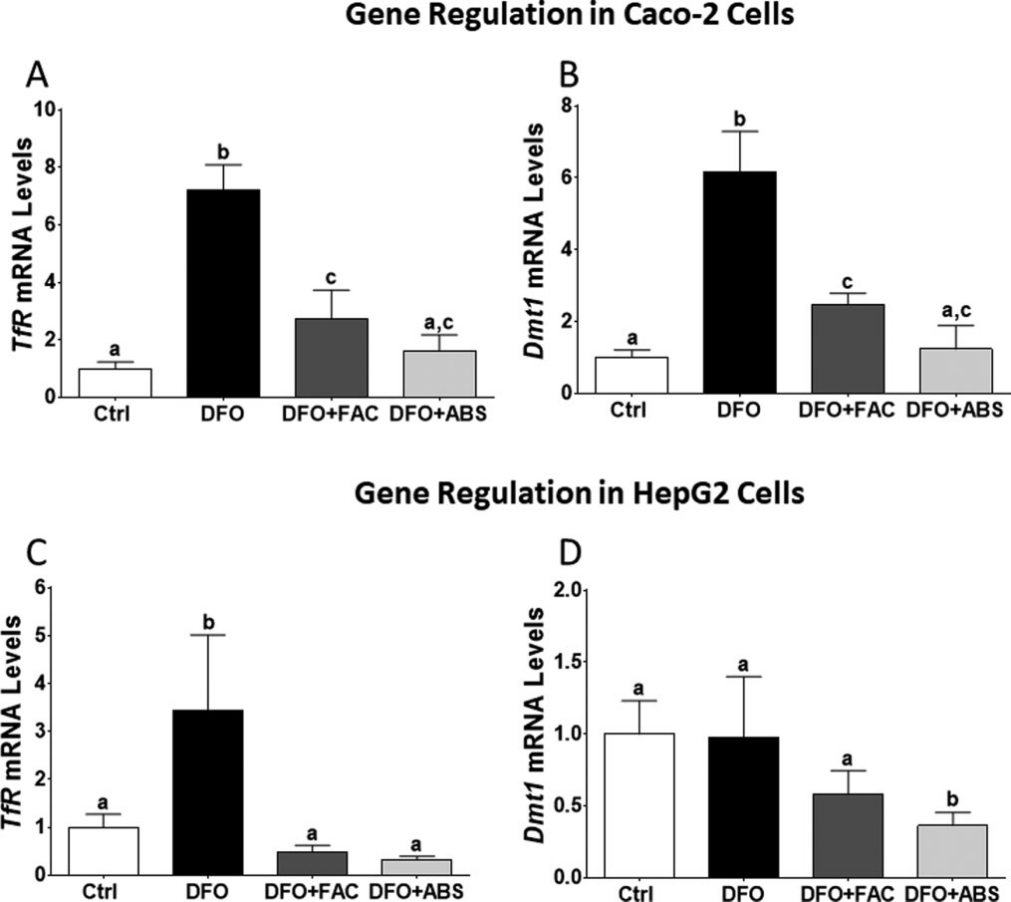
Messenger RNA levels of iron-regulated genes in Caco-2 and HepG2 cells. Total RNA was isolated from experimental groups following quantitative reverse transcription polymerase chain reaction (RT-qPCR) which was performed to analyze iron-regulated gene expression (Caco-2 cells: A and B; HepG2 cells: C and D). Different letters show statistical significance between groups within each panel. Letters depict mean (standard deviation); n = 4 independent experiments, *P* ≤ .05. *TfR* indicates transferrin receptor; *Dmt1*, divalent mineral transporter 1.

**Figure 2.**
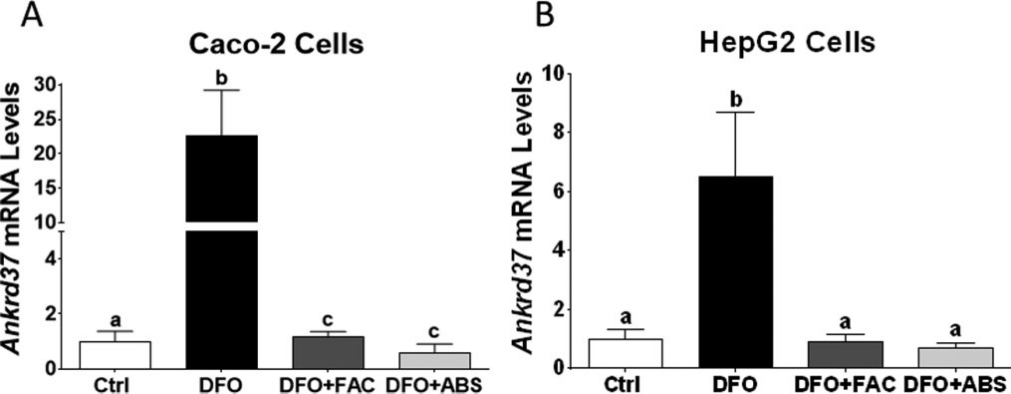
Effect of ABS on hypoxia-regulated *Ankrd37* gene in Caco-2 and HepG2 cells. *Ankrd37* is an iron-regulated hypoxic gene and mRNA levels of *Ankrd37* were analyzed by RT-qPCR (Caco-2 cells: A; HepG2 cells: B). Different letters show statistical significance between groups within each panel. Letters depict mean (standard deviation); n = 4 independent experiments, *P* ≤ .05. *Ankrd37* indicates ankyrin repeat domain 37; ABS, Ankaferd Blood Stopper; RT-qPCR, quantitative reverse transcription polymerase chain reaction.

**Figure 3.**
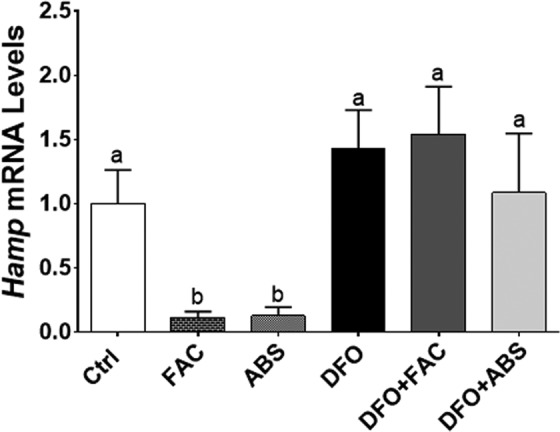
Regulation of *Hamp* gene by ABS with or without iron-deficiency anemia. Hepcidin is small peptide that controls intestinal iron absorption and is produced by the liver. *Hamp* mRNA levels were determined by RT-qPCR. Different letters show statistical significance between groups within each panel. Letters depict mean (standard deviation); n = 4 independent experiments, *P* ≤ .05. *Hamp* indicates hepcidin; ABS, Ankaferd Blood Stopper; RT-qPCR, quantitative reverse transcription polymerase chain reaction; HAMP, hepcidin protein.

Iron level was measured 7.7 µg/mL in ABS solution. We did not observe any dead cells due to ABS, FAC, and DFO treatments in our experimental models. The DFO treatment significantly induced *TfR* and *Dmt1* messenger RNA (mRNA) expression levels in Caco-2 cells (Figure 1A and B). The DFO increased *TfR* mRNA level in HepG2 cells; however, *Dmt1* mRNA level was not affected by DFO treatment (Figure 1C and D). Both cells were treated with ABS in order to understand whether the effect of iron in ABS solution decreased mRNA expression levels of these genes. We found that ABS significantly reduced *TfR* and *Dmt1* mRNA induction in Caco-2 cells (Figure 1A and B). Moreover, ABS also reduced *TfR* mRNA induction and the basal level of *Dmt1* mRNA level in HepG2 cells (Figure 1C and D). Ferric ammonium citrate treatment showed very similar results that we observed from ABS treatment in terms of *TfR* and *Dmt1* mRNA regulation in both cell lines under DFO-induced IDA (Figure 1). However, ABS significantly reduced basal level of *Dmt1* mRNA relative to FAC treatment in HepG2 cells (Figure 1D). Next, possible role of ABS on iron-deficiency-induced hypoxia was studied by measuring mRNA level of hypoxia-regulating *Ankrd37* gene.^16,18^ The DFO treatment significantly increased *Ankrd37* mRNA expression in Caco-2 and HepG2 cells. Both FAC and ABS decreased induction of *Ankrd37* transcription (Figure 2A and B). Iron is an important nutrient for *Hamp* gene regulation in liver, and HAMP protein is a key systemic regulator for iron metabolism in human.^14^ Thus, we tested whether FAC and ABS had effect on *Hamp* gene regulation with or without DFO-induced IDA in HepG2 cell. Results indicated that FAC and ABS treatments significantly reduced *Hamp* gene mRNA expression, and this reduction in *Hamp* mRNA was reversed by DFO (Figure 3). Moreover, DFO treatment did not affect *Hamp* mRNA regulation in HepG2 cells.

## Discussion

In vitro and in vivo ABS-related studies including bleeding,^19^ different types of cancer,^20^ wound healing,^21^ and inflammation are of great interest.^22^ The ABS has ability to form impermeable contact between red blood cells via modulation of ankyrin, spectrin, and actin proteins with fibrinogen gamma.^23^ It has been purposed that iron can bind to fibrinogen and this interaction might play a role in the function of ABS on tissue bleeding.^24^ Recently, Akar et al indicated that ABS had significant amount of iron and very low concentration of copper and zinc.^7^ Thus, we investigated whether ABS-derived iron can ameliorate DFO-induced iron deficiency in vitro. We used human Caco-2 and HepG2 cell lines as models of this study. After Caco-2 cells are grown at 21 days postconfluent, they show physiological similarities with human enterocyte cells.^16^ Enterocyte cells of intestine are main contributor to arrange iron balance in human. Moreover, liver is also important for iron homeostasis in terms of iron storage.^10^ *TfR* and *Dmt1* are used as marker genes to evaluate whether ABS-derived iron reduced effect of IDA. *TfR* and *Dmt1* are very responsive to iron deficiency in vitro and in vivo.^13,16^ Results indicated that DFO significantly increased mRNA levels of *TfR* and *Dmt1* genes, and this induction was reversed by FAC and ABS. However, DFO did not have an effect on *Dmt1* mRNA levels in HepG2 cells. This result was consistent with the literature.^25^ This might be related to liver iron metabolism during anemia, because transferrin-transferrin receptor (TFR)-dependent endocytosis is one of the main molecular mechanisms for iron absorption during anemia in liver.^26^ Induction of *TfR* genes in this study might explain TFR-dependent regulation of iron uptake in HepG2 cells.

It has been recently shown that hypoxia is a key molecular signal for iron metabolism.^11^ Iron deficiency causes cellular hypoxia via increasing protein stability of hypoxia inducible factor 1α (HIF1α) and HIF2α transcription factors due to inactivation of HIF prolyl-hydroxylases (PHDs), but available iron can reduce stability of HIF1α and HIF2α by activation of PHD enzymes.^27^ *Ankrd37* is one of the most sensitive genes that is regulated by hypoxia.^16,18^ Thus, *Ankrd37* was used as a genetic marker for hypoxia in the current study. Data showed that DFO induced transcription of *Ankrd37* gene, whereas FAC and ABS decreased induction of this gene. These results suggest that ABS-derived iron has ability to reduce iron-deficiency-induced hypoxia in both cell lines. Next, the effect of ABS-derived iron was investigated on transcriptional regulation of *Hamp* gene in HepG2 cells. It has been indicated that increased levels of iron in blood leads to induction of *Hamp* gene.^28^ Surprisingly, both FAC and ABS reduced *Hamp* mRNA expression without anemia. It has been indicated that *Hamp* mRNA expression was suppressed by iron loading in HepG2 cells,^29,30^ and this is consistent with the current data. It was suggested that systemic factor(s) was required for in vitro regulation of *Hamp* gene.^30,31^ The ABS-related studies have been dramatically increased in different scientific areas. Possible effects of iron in ABS should consider non-iron-related studies because iron has a significant effect on gene regulation in vitro and in vivo.^13,16,32^ Thus, ABS-derived iron might be a contributor to molecular and genetic studies.

## Conclusion

Our results showed that FAC and ABS had very similar effects on gene regulation of anemic Caco-2 and HepG2 cells. This suggests that ABS-derived iron is biologically active to reduce IDA. It has been indicated that plant-based molecules can reduce bioavailability and biofunctionality of iron.^9^ Our results showed that plant-based molecules in ABS did not interfere with the function of iron in cells. ABS-derived iron might help to decrease anemia by providing iron when ABS stops tissue bleeding. This is the first in vitro study to investigate the effect of ABS on anemia. However, animal and human studies are necessary to clarify the effect of ABS-derived iron on IDA.
